# Current global population size, post-whaling trend and historical trajectory of sperm whales

**DOI:** 10.1038/s41598-022-24107-7

**Published:** 2022-11-14

**Authors:** Hal Whitehead, Megan Shin

**Affiliations:** Department of Biology, Dalhousie University, Halifax, NS B3H 4R2 Canada

**Keywords:** Ecology, Zoology, Ocean sciences

## Abstract

The sperm whale lives in most deep ice-free waters of the globe. It was targeted during two periods of whaling peaking in the 1840’s and 1960’s. Using a habitat suitability model, we extrapolated estimates of abundance from visual and acoustic surveys to give a global estimate of 736,053 sperm whales (CV = 0.218) in 1993. Estimates of trends in the post-whaling era suggest that: whaling, by affecting the sex ratio and/or the social cohesion of females, reduced recovery rates well after whaling ceased; preferentially-targeted adult males show the best evidence of recovery, presumably due to recruitment from breeding populations; several decades post-whaling, sperm whale populations not facing much human impact are recovering slowly, but populations may be declining in areas with substantial anthropogenic footprint. A theta-logistic population model enhanced to simulate spatial structure and the non-removal impacts of whaling indicated a pre-whaling population of 1,949,698 (CV = 0.178) in 1710 being reduced by whaling, and then then recovering a little to about 844,761 (CV = 0.209) in 2022. There is much uncertainty about these numbers and trends. A larger population estimate than produced by a similar analysis in 2002 is principally due to a better assessment of ascertainment bias.

## Introduction

The sperm whale *Physeter macrocephalus*, is the largest of the toothed whales. It is a deep diver, and uses most of the ice-free waters of the globe greater than 1000 m deep^1^. Sperm whales are thought to be important elements in marine ecosystems because of their numbers, size, wide-distribution, and ability to recycle nutrients from the depth to the photic zone^2,3^. The hunting of sperm whales was economically significant and global in scale, and used two principal methodologies^4,5^. Killing from open boats began in 1712, peaked in about 1840, and continued into the 1920’s. ‘Modern’ whalers, with larger engine-powered whaling vessels, harpoon guns and other technological aids, first targeted sperm whales in the early twentieth century, although the hunt became much more intense between 1950–1970^6^. Following the International Whaling Commission’s moratorium on commercial whaling in 1986 there has been very little whaling for sperm whales (an average of 12 whales per year^7^.

In 2002, one of us produced a global estimate of sperm whale population size in year 2000 by extrapolating estimates from visual surveys of about 24% of sperm whale habitat^8^. This estimate (360,000 whales; CV = 0.36) was then used together with catch data to construct a historical trajectory of sperm whale populations. This population estimate and trajectory have been widely cited, despite the paper stating: “the global estimates may be more imprecise than is suggested by the calculated CV” and the “CV itself is somewhat disappointingly large”^8^.

Here, 20 years later, we update and improve the global population estimate and trajectory, as well as reviewing results on trends in population size since the end of whaling. Improvements include: the inclusion of a number of more recent surveys, some of new areas, and usually using more developed field and analytical methodology^9^; better consideration of the correction for availability bias, the probability that a whale on the survey line is counted, *g*(0)^10^; the inclusion of acoustic surveys^11,12^; the addition of habitat suitability modelling to better extrapolate from surveyed areas to a global estimate^13,14^; a more considered estimate of the potential maximal rate of increase^15^; new information on historical catch data^6,16,17^; incorporation of spatial structure into the population model^18^ as well as the demographic consequences of social disruption caused by whaling^19^.

Additionally, we examine trends in sperm whale since the end of substantial whaling in about 1983 (when catches fell below 500 per year^6^). Sperm whale abundance would be expected to have increased with the end of significant removals from a depleted population due to density-dependent effects, but some analyses have found no indication of this, perhaps due to the lingering non-removal effects of whaling on sperm whale demography, and/or the effects of more recent threats such as ocean noise, ship strikes and entrapment in fishing gear^19–24^. We review such studies to suggest global post-whaling trends.

The primary goals of this paper are to estimate: the current (2022) size of the global sperm whale population, the pre-whaling (1711) population, the three-generation decline (IUCN criterion for listing^25^), post-whaling trends in population size, and to plot an estimate of the population’s historical trajectory.

## Results

### Surveys

**Figure 1:**
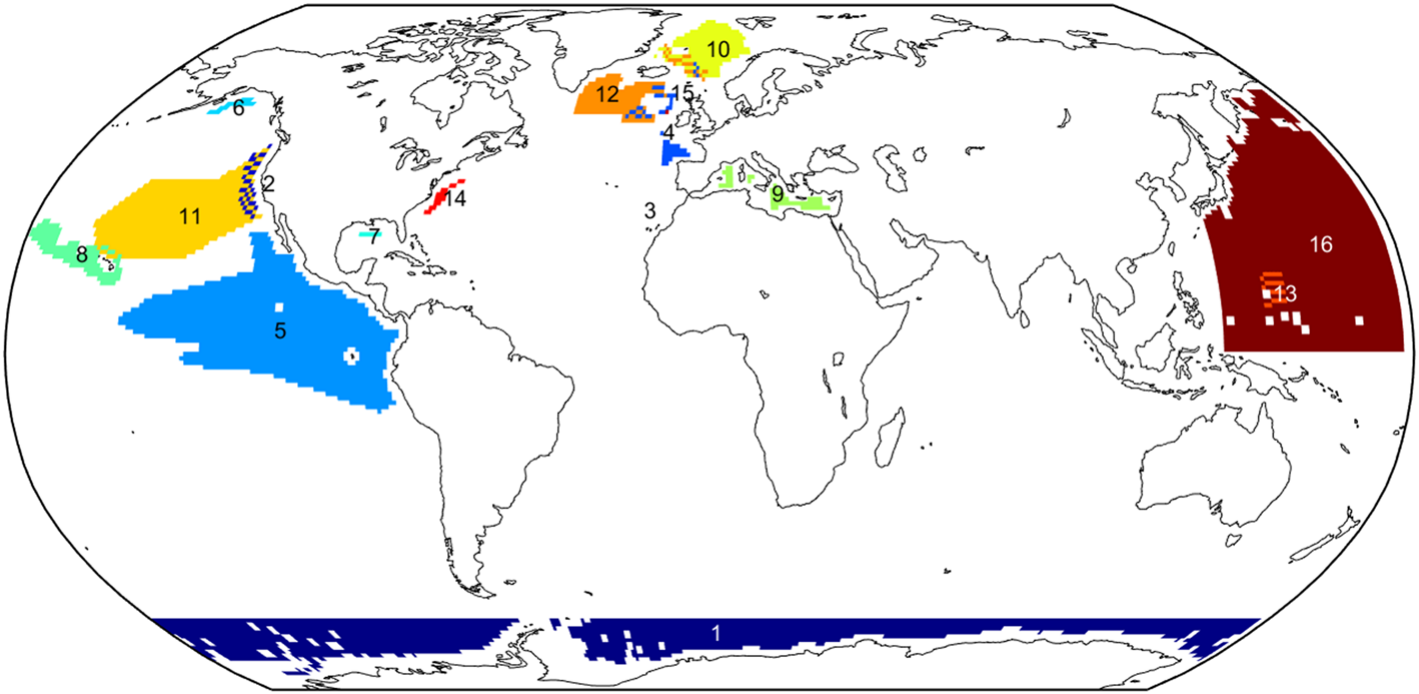
Sperm whale surveys used to produce global population estimates. Numbers by each colour reference surveys listed in Table 1. Where surveys overlap the colours are pixelated. Unsurveyed waters, waters less than 1000 m deep and land are uncoloured. Map created using MATLAB2021b (mathworks.com).

Surveys which estimate sperm whale population size are listed in Table 1 and mapped in Fig. 1. They cover a total of 73,681,588 km^2^, 24.0% of the global sperm whale habitat (306,594,122 km^2^), with 0.8% of the global habitat included in two different surveys (see Fig. 1).

**Table 1:** Population estimates for sperm whales used to construct global population estimate.

	Region	Years	Type	*g*(0)	Area 1000 km^2^	Population	CV	Whales/1000 km^2^	Source
1	Antarctic	1985–1991	Ship visual	*0.60*	15,234	17,500	0.33	1.15	^26^
2	California current	2014	Ship visual	Varied	859	2281	0.57	2.66	^20^
3	Canary islands	2009–2010	Ship acoustic	0.92	33	224	0.32	6.88	^27^
4	Eastern North Atlantic	2005–2007	Ship visual	*0.60*	1086	5445	0.37	5.02	^28^
5	Eastern tropical Pacific	1986–1990	Ship visual	*0.60*	18,166	37,777	0.37	2.08	^29^
6	Gulf of Alaska	2015	Ship visual	*0.60*	266	575	0.52	2.16	^30^
7	Gulf of Mexico	2017–2018	Ship visual	*0.60*	209	1967	0.36	9.41	^31^
8	Hawaiian islands	2017	Ship visual	0.62	2126	5095	0.56	2.40	^32^
9	Mediterranean sea	2003–2013	Ship acoustic	0.92	1093	1842	0.23	1.69	^12^
10	Northeast Atlantic	2002–2007	Ship visual	*0.60*	1494	13,557	0.34	9.07	^33^
11	Northeast Pacific	1997	Ship acoustic	1.00	7670	32,068	0.36	4.18	^34^
12	Northern Atlantic	2007	Ship visual	0.57	2111	12,268	0.33	5.81	^35^
13	Northern Mariana islands	2007	Ship visual	*0.60*	563	1175	0.67	2.09	^36^
14	Northwest Atlantic	2016	Ship and aerial, visual	0.61	412	4349	0.28	10.55	^37,38^
15	Offshore Ireland	2015–2016	Ship acoustic	1.00	45	380	0.04	8.43	^11^
16	Western North Pacific	1982–1996	Ship visual	*0.60*	24,752	43,027	0.31	1.74	^39^

The correction for availability bias (i.e. whales being under water), *g*(0), is as given in the paper or, if not given, the assumed generic value (*italics*). The survey areas exclude waters less than 1000 m deep.

As sperm whales are more easily heard than seen, acoustic surveys generally needed very little correction for availability bias. In contrast, all visual surveys were corrected using *g*(0) either by the researchers themselves, or, for the 8 surveys where this was not done, using a generic default value of *g*(0) = 0.60.

### Habitat suitability

The relationships between 14 predictor variables and sperm whale density across the different surveys are shown in Supplementary Fig. S1, together with linear and quadratic best fit regressions. No variable is an extremely good predictor of sperm whale density, but several (such as depth, salinity, phosphates and nitrates) seem to show a relationship.

Stepwise regression of sperm whale density from each survey on the predictor variables (weighting by survey area) retained three variables, without including any quadratic terms. The retained predictor variables were salinity, average nitrogen and depth (expressed as a negative number relative to the surface):

$${\text{Predicted density }} = \, 0.0{712 } - \, 0.00{166} \cdot {\text{Salinity }}{-} \, 0.000{255} \cdot {\text{Nitrates }} + \, 0.00000{151} \cdot {\text{Depth}}$$

Thus, the prediction is that sperm whales are more abundant when waters are lower in salinity and nitrates, and shallower (but more than 1000 m deep). This relationship has an adjusted R^2^ = 0.561, indicating some predictive ability. A similar analysis weighting all surveys equally rather than by area included most predictor variables and was consequently overfitted.

**Figure 2:**
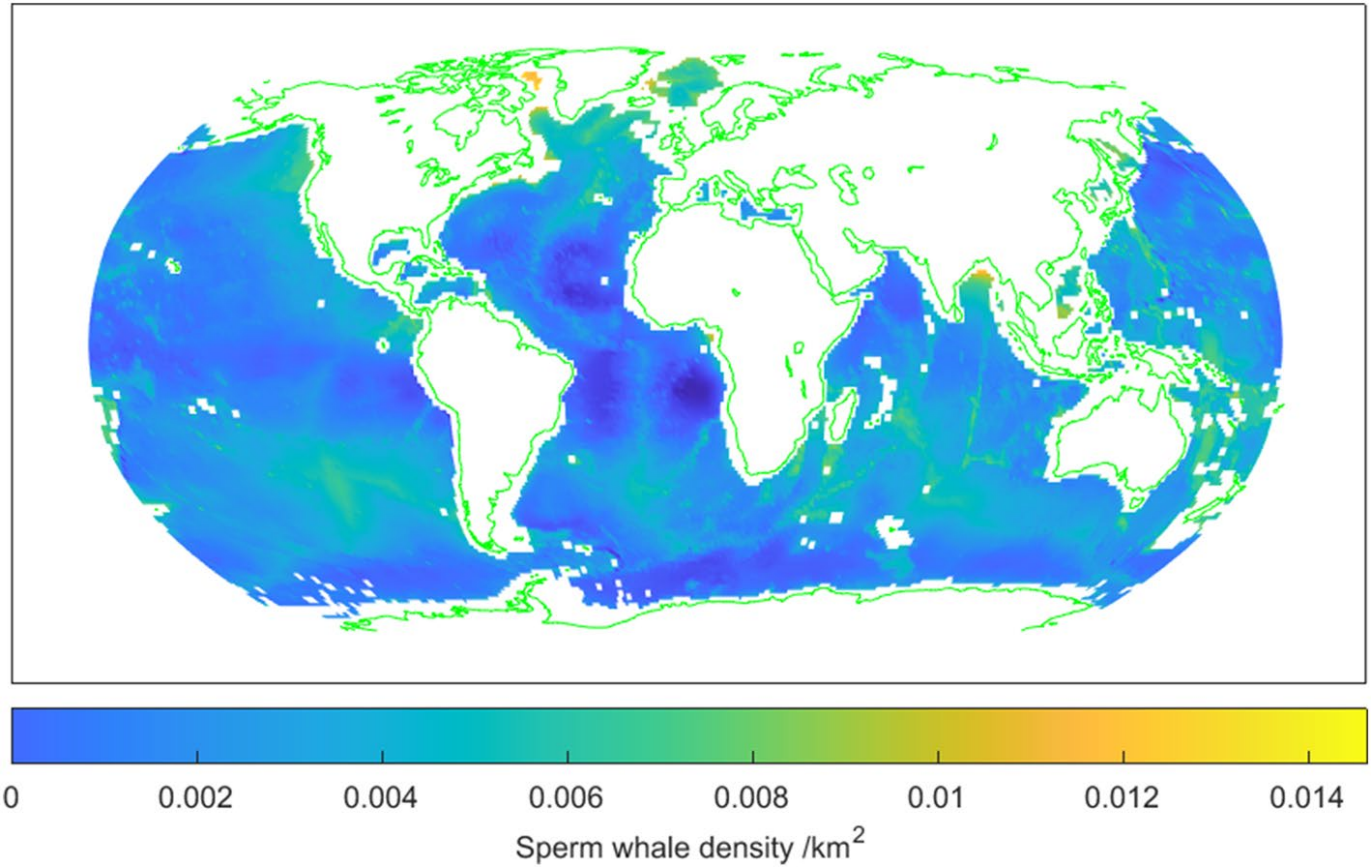
Predicted density of sperm whales from weighted stepwise regression of survey densities on oceanographic variables. Waters less than 1000 m deep and land are uncoloured. Map created using MATLAB2021b (mathworks.com).

The predicted global distribution of sperm whales using the weighted stepwise regression is shown in Fig. 2. The plot seems mostly reasonable (for instance it is generally similar to the pattern in Fig. 8.2.38 of^40^ which was produced using very general and largely qualitative classes of habitat preference). However, there are some small-scale anomalies, such as some high predicted densities in the high Arctic, and negative densities off west Africa.

### Densities and populations

Estimated sperm whale densities in the survey areas varied from 1.15 whales/1000 km^2^ in the Antarctic to 10.55 whales/1000 km^2^ off the US continental shelf in the northwest Atlantic (Table 1). The mean density over all survey areas was 2.31 whales/1000 km^2^ (CV = 0.137).

Extrapolating these densities to the global sperm whale habitat by area gives an estimated 706,932 whales at a density of 2.31 whales/1000 km^2^ (CV for both density and population = 0.218) in 1993. The year 1993 is the mean year of the surveys in Table 1, weighting each survey by the area of its study area. Extrapolating using the oceanographic predictor variables gives 736,053 whales globally at a density of 2.40 whales/1000 km^2^ (CV = 0.218). Estimates for the North Atlantic, North Pacific and Southern Oceans are presented in Table 2.

**Table 2:** Population and density estimates for sperm whales in different ocean areas in 1993, extrapolating from survey data using weighted stepwise regression of survey densities on oceanographic variables.

Ocean	Area 1000 km^2^	Population	CV	Density/1000 km^2^
Global	306,594	736,053	0.22	2.40
North Atlantic	37,229	92,085	0.38	2.47
North Pacific	72,160	177,555	0.20	2.46
Southern Hemisphere	187,984	443,311	0.23	2.36

The equator is a latitudinal boundary of the ocean areas.

### Post-whaling trends in population size

**Figure 3:**
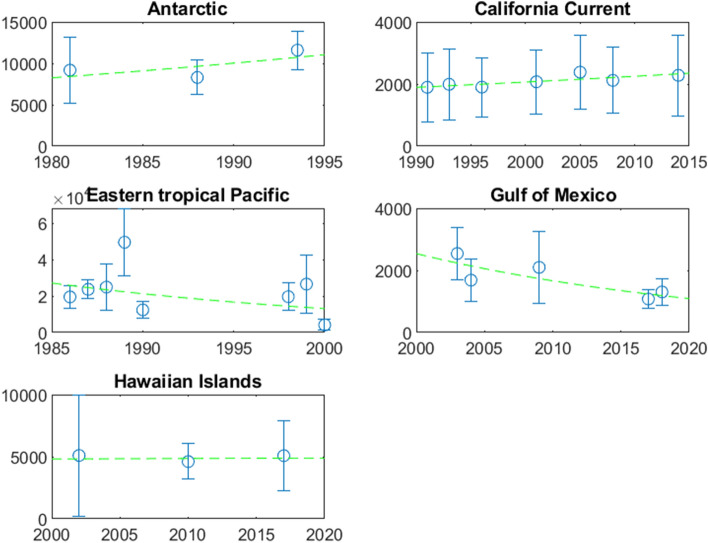
Trends in the estimated populations of sperm whales in five study areas where repeated surveys covered at least 10 years and there were at least 3 such surveys. Error bars indicate standard errors, and trend lines are shown (see Table 3 for further details).

The estimated population trends across five sets of visual surveys are listed in Table 3, and illustrated in Fig. 3. Rates of increase vary from − 0.049/yr in the eastern tropical Pacific to + 0.019/yr in the Antarctic. We had originally planned a meta-analysis of these results using mixed or Bayesian hierarchical models, but the data sets tell of several scenarios. Thus, they are not replicate studies of the same phenomenon, and not suitable for such analyses. Here is a synopsis of the scenarios:

**Table 3:** Studies used in post-whaling trend analysis.

Region	Years	Data	Method (years of data)	Area 1000 km^2^	Trend/yr	SE	Source
Antarctic	1978–1998	Ship visual	Abundance estimates (3)	15,234	0.019	0.022	^26^
California current	1991–2014	Ship visual	Abundance estimates (7)	859	0.009	0.003*	^20^
Eastern tropical Pacific	1986–2000	Ship visual	Abundance estimates (8)	18,166	− 0.049	0.043	^41^
Gulf of Mexico	2003–2018	Ship visual	Abundance estimates (5)	209	− 0.042	0.015	^31^
Hawaiian islands	2002–2017	Ship visual	Abundance estimates (3)	2126	0.001	0.009	^32^
Galápagos	1985–1995	Ship sightings	Sighting rates (6)	~ 65	− 0.090	0.086*	^19^
Galápagos	1985–1995	Photo-identifications	Mark-recapture (9)	~ 65	− 0.200	0.064^†^	^19^
Guadeloupe	2001–2013	Photo-identifications	Mark-recapture (13)	~ 1.4	− 0.062	0.030^†^	^42^
Dominica-Guadeloupe	2005–2015	Photo-identifications	Count of individuals (10)	~ 4.6	− 0.195	0.059^†^	^43^

*Calculated using weighted regression from presented data.

^†^Calculated from presented confidence interval.

The eastern tropical Pacific analysis indicates a population decline of about 4%/yr over a large area in the years following the end of whaling (1981 in Peru^44^), although there is uncertainty. The mark-recapture and sighting estimates from the Galápagos Islands, in the heart of the eastern Tropical Pacific, also show declines during that period, although some of the changes were likely caused by redistribution of the whales^19^. However, these declines, together with low calving rates and few large males off the Galápagos in the years following the end of whaling^19^, provide evidence for the demographic effects of social disruption caused by whaling. They also indicate the scale—around 5% per year—and duration—perhaps 15 years—of such effects, which are incorporated in the parameterizing of the population model (Table 4).

**Table 4:** Parameters of population model.

Parameter	Explanation	Best value	Range for sensitivity analysis	Assumed distribution	Source/Comments
*n*(1993)	Population in year 1993	736,053	422,995–1,049,111	Normal (CV = 0.218)	Extrapolation from surveys
*r*	Maximum potential rate of increase	0.0096/yr	0.0046–0.0146	Uniform [0.0046–0.0146]	^15^
θ	Shape of density-dependent function	2.39	1.0–18.17	Taken from {1.00, 1.55, 2.39, 4.55, 18.17}	^45^Adding intermediate (on a log scale) values of 1.55 and 4.55
*f*(1712–1899)	Correction for earlier catch	1.1	1.05–1.15	Uniform [1.05–1.15]	^17^
*f*(1900–1986)	Correction for later catch	1.05	1.01–1.20	Uniform [1.01–1.20]	Based on^6^
*K*	Number of subpopulations	8	1–15	Taken from 1:15	*K* = 10 and α = 0.7 gave an expansion of whaling efforts to new populations in the early 1800’s in line with the historical record
α	Proportion of original size when exploitation moves to next population	0.7	0.5–0.8	Uniform [0.5–0.8]	
*q*	Reduction in rate of increase due to social disruption of whaling	0.05/yr	0.0–0.1	Normal (SE = 0.02)	Based upon decrease in Eastern Tropical Pacific post whaling
*T*	Number of years over which social disruption occurs	15 yr	2–25	Taken from {2, 4, 8, 16, 32}	Based upon decrease in Eastern Tropical Pacific post whaling. Effects documented in elephants^46^

In contrast, the time series for the California Current and Hawaiian Islands indicate a slow increase in more recent years. These estimated rates of increase are within 1SD of the estimated maximum potential rate of increase for a sperm whale population of about 1% per year^15^. These study areas are more removed from intense whaling than the whales in the eastern tropical Pacific, both by later starts of the survey time series (1991 and 2002), and by an earlier end to intense whaling in the central and eastern North Pacific (early 1970’s). Thus, the indication is that about two decades after the end of whaling the demographic consequences of social disruption have largely dissipated.

The clearest sign of a post-whaling increase is from the Antarctic, where there are only mature males as females and immature males are restricted to latitudes less than about 50^o^^1^. This increase would be expected as mature males were the most heavily affected by modern whaling, and recruitment into their depleted polar habitat from less affected low-latitude breeding populations should lead to a rebound following the end of whaling^19^. Unfortunately, we do not have good trend data for any other largely-male habitat.

In the smaller study areas, the Gulf of Mexico and eastern Caribbean, ship surveys and mark-recapture studies indicate declines in abundance since 2000 (Table 3). This suggests that in nearshore areas with substantial anthropogenic impact, sperm whale populations are, in current times, in some jeopardy.

However, as a general caution, we stress that the estimated trends indicated in Table 3 are mostly either very small or very uncertain, or both.

### Historical trajectory

**Figure 4:**
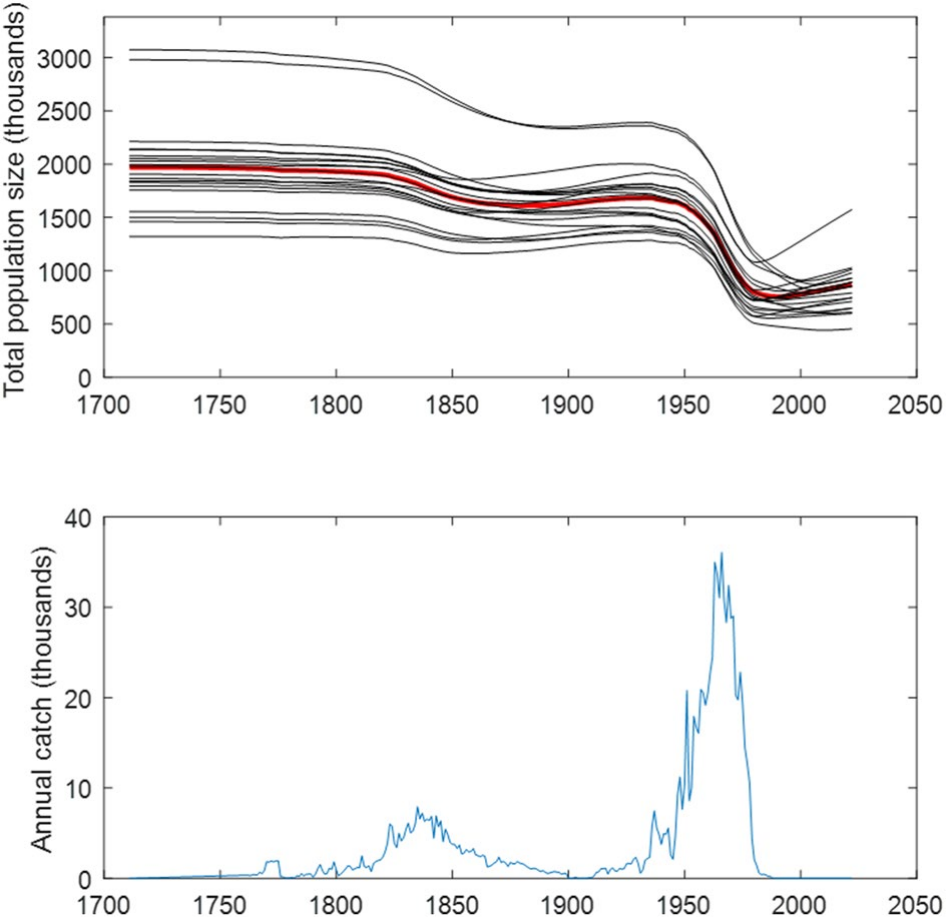
Upper: Estimated trajectories of global sperm whale population using best estimates for model parameters in red, and 20 trajectories using randomly chosen but reasonable parameter estimates in black. Lower: Reported annual global sperm whale catch.

The historical trajectory of the global sperm whale population using the 1993 estimate extrapolated from surveys, the annual reported catches, and the best estimates of population parameters is shown in Fig. 4, along with twenty of the trajectories with randomly-chosen parameter values. (Eight of the 1000 runs with randomly-chosen parameter values gave invalid 1711 population estimates.) Declines during the two major phases of sperm whaling, peaking in the 1840’s and 1960’s, are clear, and a recovery beginning in the 1990’s is indicated. The variety of population trends in the 21st Century in the random-parameter trajectories shows our uncertainty about this recovery. Depending on the length and nature of the effects of social disruption caused by whaling, the current trend can be positive—if social cohesion has recovered or was not greatly affected by whaling—or only just beginning to level out—if the social disruption of whaling had a major and long-lasting effect on sperm whale demography.

Using the best parameter estimates, with the random parameter runs providing confidence intervals, the population in 1711 is estimated as 1,949,698 (SE = 335,054; CV = 0.178; 95% CI 1,296,811–2,575,057; see Supplementary Fig. S2). In 2022, the estimated population is 844,761 (SE = 171,206; CV = 0.209; 95% CI 481,901–1,153,459). The three-generation decline (1940–2022) is estimated as 0.4819 (SE = 0.1035; CV = 0.215; 95% CI 0.2631–0.6696; see Supplementary Fig. S2). The estimated overall decline from 1711–2022 is 0.5667 (SE = 0.1172; CV = 0.212; 95% CI 0.2843–0.7555).

The sensitivity analysis (Supplementary Fig. S3) indicates that these estimates and the trajectory are most affected by the estimate of 1993 population size, *n*(1993), as by well as the significance and duration of social disruption caused by whaling (*q* and *T*), and least by the catch corrections (*f*).

## Discussion

We estimate a global population of 736,053 sperm whales at a density of 2.40 whales/km^2^ (CV = 0.218) in 1993. The CV, assessing confidence in this estimate, includes uncertainty due to sampling issues in each population survey, uncertainty about the availability bias, *g*(0), and extrapolation from sampled areas to the global habitat for sperm whales. The elements of the CV estimate are themselves uncertain, but not obviously biased.

However, there are some potential sources of error and bias not included in the population and CV estimates. Perhaps the most serious is that the survey areas are largely in the northern hemisphere (with the principal exception of the Antarctic, unique sperm whale habitat). In the extrapolation process we assume that variation in sperm whale density among study areas in the northern hemisphere is representative of differences between the hemispheres in sperm whale habitat suitability, given the oceanographic variables that we included. As different ocean basins have fundamental contrasts in their oceanography, this may be an optimistic assumption. But, for now, it is untested.

Two other issues are probably less serious. First, we excluded waters less than 1000 m deep, even though sperm whales, especially male sperm whales, are known sometimes to use such waters^47^. However, densities are typically lower than in deeper waters^37^, and the global area of such habitat (roughly 400–1000 m deep) is low compared with deeper waters. Secondly, the different surveys are treated as independent samples to obtain the habitat suitability prediction function used to extrapolate globally, but there is some overlap. However, only 3.6% of the grid points used were included in two surveys (none in three or more), so, given the overall uncertainty in the global population estimate, the effect of double counting is likely insignificant.

Compared with the earlier population estimate for year 2000^8^, our estimate for 1993 is 96% higher (706,932 vs. 361,400). A number of the surveys used to estimate populations, especially the larger and more heavily-weighted ones, are in both studies. The great difference is that the default availability bias for visual ship surveys was assumed to be *g*(0) = 0.87 in^8^, while several more recent estimates consistently suggest *g*(0) = 0.60. If applied to Whitehead’s^8^ global estimate, *g*(0) = 0.60 would have given an estimated population of 524,030, 1.3 SDs lower and so not significantly different (at P < 0.05) from our current 1993 estimate. There are other potential causes of the higher estimate in this paper. Even though the overall survey coverage is not changed from the surveys used by^8^ (24% of global sperm whale habitat), the more recent estimates indicate higher densities, perhaps because of better survey techniques. Additionally, the extrapolation to global habitat suitability using environmental variables suggests that the surveyed areas were generally slightly less suitable habitat than the oceans as a whole.

There are several ways to improve the estimate of recent sperm whale abundance. Improving the survey coverage, especially in the southern hemisphere, is most obvious. While visual surveys have been the primary source of abundance estimates (Table 1), they are logistically and financially demanding, especially in waters far from land. Acoustic surveys are becoming more common, and procedures for analysis have been developed in recent years so that they seem to produce more precise estimates than visual surveys with comparable effort^34^. Methods for surveying acoustically from autonomous vessels and drifting buoys are under development^48,49^ and these should considerably reduce costs and the feasibility of large-scale acoustic surveys in a wide range of habitats and conditions. Satellite imagery is another potential solution to the problem of surveying far offshore^50^. However, unless there is a full global survey, there needs to be extrapolation to unsurveyed areas. Here we used habitat suitability mapping to make this extrapolation, but it was carried out in a rather crude way, considering population survey areas (of very varied sizes) as independent units. There has been more sophisticated fine-scale habitat mapping of sperm habitat in some, relatively small and usually near-shore, ocean areas^13,14,51^, but the results are not consistent. Larger-scale habitat studies, including offshore waters in different oceans, and including environmental variables which might be more relevant for sperm whales (e.g. measures of the abundance of cephalopods, the primary sperm whale prey), could make for a more satisfactory extrapolation to unsurveyed areas.

Our review of studies of trends in sperm whale populations over the roughly 40 years since the end of whaling did not identify a single clear trend. Instead, several processes seemed to be affecting the population dynamics of the sperm whales now largely free from the whaling of the preceding 270 years: that whaling causes social disruption and other lingering effects which can reverse the expected population increase following exploitation; that such declines may be of the order of − 5%/yr and last for a decade or two; that, once these effects have run their course, sperm whale populations largely free from other adverse impacts may increase at about the maximum theoretical rate of increase, + 1%/yr^15^; that populations of adult male sperm whales, which were particularly targeted by modern whalers, have, since the end of whaling, been increasing more quickly than the general population; and that in areas of substantial anthropogenic impact sperm whale populations may be declining.

The support for these processes is not very strong. They are mostly based on one or two estimates of trend, most of which are not statistically significantly different from zero at P < 0.05. With their very low reproductive rates, in the absence of substantial immigration positive trends in sperm whale populations are constrained to be very low, with the exception of mature male populations recovering from near eradication through recruitment. There are indications of such increases for males in the Antarctic and California Current^20,26^, but no sign of recovery in the population of large male sperm whales off southwestern Australia between surveys in 1968–1978 and 2009^21^. Declines can be larger in magnitude than increases, and the largely independent estimates of decline in the eastern Caribbean are concerning^42,43^, as is that in the Gulf of Mexico^31^. The nearly isolated population of sperm whales in the Mediterranean Sea is also believed to be at risk^52^, although no good trend data are currently available.

We are now nearly 40 years since the end of whaling and the opportunity for learning more about how sperm whale populations responded to whaling’s immediate aftermath has largely gone. Moving forward, we can obtain a clearer view of current trends using the methods currently available—ship surveys, mark recapture analyses, acoustic recordings—preferably with greater intensity, wider geographic and temporal scope and standardized methodology, as well as new methods, such as acoustic surveys from drifters and autonomous vehicles as well as satellite images.

Our population model gives estimates of the current, 2022, population (844,761), the initial, 1711, population (1,949,698), the overall, 1711–2022, decline (57%), and the 3-generation, 1940–2022, decline (48%). All these estimates have a similar proportional error with a CV ~ 0.2. This level of error is estimated from reasonable (to us) estimates of uncertainty in the input parameters.

However, not included is uncertainty about the form of model. While the theta-logistic is often used as a default population model^53^, there are alternatives. However, as variation in the parameters *r* and θ include most realistic forms of density dependence, and sperm whales do not seem to have reached the very low abundance levels where inverse density dependence (the Allee effect) can come into play and complicate the recruitment-abundance relationship, the theta-logistic seems a reasonable model, and its innate inaccuracies are likely dwarfed by those in the input parameters. However, variation in carrying capacity (*n*(1711) in our formulation) is a potentially important issue^54^. There seems to have been considerable variation in sperm whale population size over evolutionary time^55^, which might indicate that there is also variation in global carrying capacity over the ecological time scales considered here.

We made two modifications to the theta-logistic to mimic seemingly important elements of sperm whale population structure: spatial structure and the demographic consequences of the social disruption caused by whaling. There is very strong evidence for spatial structure^18^ and good evidence for social disruption having population-level effects. However, the realism of our modelling of these effects is less clear. Spatially, it would be better to use the actual geographical structures of sperm whales and whaling (e.g. ocean basins), for which we would need a breakdown of catches by area for each year, which is not currently available for the entire whaling time series. Sperm whale populations seem more structured by culture than geography and ideally should be managed at the level of the cultural clan^56^. However, we have no way to break down catches by clan, so this seems impossible. A linear decline in rate of increase with proportional catch over the previous *T* years may be a good way to model the effects of social disruption, but we don’t know this. However, we suspect, but do not know, that the simplicities of our model in these respects are less important than uncertainty in the model parameters which we do include.

Based upon the sensitivity analysis (Supplementary Fig. S3) we surmise that uncertainty about the historical trajectory of the global sperm whale population is: most strongly caused by uncertainty in the estimates of the 1993 population (*n*(1993)) and the effects of social disruption (*q*); somewhat due to uncertainty in the maximum rate of increase (*r*), the shape of the density dependent function (θ) and the duration of the effects of social disruption (*T*). Uncertainty in the catch corrections and parameters determining the spatio-temporal progression of whaling (*f*, *K*, α) seem to have lesser importance. This suggests that in order to improve the precision of the historical trajectory, effort on reducing uncertainty about post-whaling population size and the significance of social disruption will have greatest payoffs.

## Conclusion

There are about 850,000 sperm whales now, down from about two million before the start of whaling in 1712, so a decline of approximately 57% in 310 years caused by substantial open-boat whaling which peaked in the 1840’s and massive modern whaling primarily between 1950 and 1975. Time series data collected since whaling ended in the 1980’s suggest several factors. Intense whaling seems to have caused social disruption and related effects, reducing recovery rates for a decade or two afterwards. However, populations of adult males which were particularly targeted in the most recent phases of whaling show better evidence of recovery, presumably due to recruitment from less exploited breeding populations. With whaling 30–40 years in the past, some sperm whale populations in relatively undisturbed areas show modest signs of recovery, but others, living with more intense anthropogenic pressures, appear to be declining. There is considerable uncertainty about these conclusions. We need better data on the size of sperm whale populations today, as well as their trends.

## Methods

### Selection of surveys and extraction of data

We selected published surveys that produced estimates of sperm whale population size or density (see Supplementary Information for methodology; surveys listed in Table 1). We extracted: the type of survey (ship, aerial; acoustic, visual), the years of data collection; the coordinates of the boundary of the study area; the estimates of *g*(0) and CV (*g*(0)) used to correct for availability bias, if given; and an estimate of sperm whale population or density in study area with CV. From these we calculated for each survey the survey area with waters greater than 1000 m deep (typical shallow depth limit of sperm whales^3^). When no value of *g*(0) was used (8 ship visual surveys) we corrected the population/density estimate using an assumed generic value of *g*(0) and recalculated the CV to include uncertainty in *g*(0) (as in Eq. 1 of^8^). Three ship visual surveys did calculate a single *g*(0) estimate: 0.62 (CV 0.35)^32^; 0.57 (CV 0.28)^35^; 0.61 (CV 0.25)^37^. These are consistent and suggest a generic *g*(0) = 0.60 (CV 0.29), also agreeing with *g*(0) = 0.60 estimated from pooled surveys in the California Current^10^.

### Global habitat of sperm whales

To extrapolate sperm whale densities from surveyed study areas to the sperm whales’ global habitat, we created a one-degree latitude by one-degree longitude grid. We removed the following grid points as not being prime sperm whale habitat^1,3,40^: points on land or with central depths less than 1000 m; largely ice-covered points in the Beaufort Sea, and the waters north of Svalbard and Russia; the Black Sea and Red Sea both of which have shallow entrances that appear not to be traversable by sperm whales.

Generally, food abundance is a good predictor of species distribution. However, this is not possible for sperm whales as we have no good measures of the abundance or distribution of most of their prey, deep-water squid^57^. Instead, oceanographic measures have been used to describe sperm whale distributions over various spatial scales with a moderate level of success^13,14^. We follow this approach. Measures that might predict sperm whale density were collected for each grid point, some at just the surface, others at the surface, 500 m depth, 1000 m depth or an average of the measures at the different depths (Supplementary Table S2). Water depth was the strongest predictor in Mediterranean encounters, when compared to slope and distance to shore^13^. Temperature and salinity have been used as predictors for the distribution of fish and larger marine animals, which could translate into prey availability and thus density for sperm whales^58,59^. Primary productivity and dissolved oxygen generally dictate the biomass of wildlife in an area, while nitrate and phosphate levels limit the amount of primary productivity in an area^60^. Eddy kinetic energy is a measure of the dynamism of physical oceanography which is becoming a commonly used predictor of cetacean habitat^61^. We did not use: latitude and longitude as these primarily describe the general geographic distribution of the study areas, and geographic aggregates of sperm whale catches^62^ as these proved to have no predictive power. The mean values of the 14 predictor measures were calculated over calendar months for each grid point, and then over the grid points in each study area.

To obtain predictors of the sperm whale density at each grid point, we then made quadratic regressions of the density of sperm whales in each study area (*i*), *d*(*i*), on the mean values of the predictor measures, weighting each study area by its surface area. Because the surveys were conducted over different time periods, the densities were corrected based on the estimated trajectory of global sperm whale populations by multiplying *d*(*i*) by the ratio of the global population in 1993 over that in the mid-year of the survey (as in Fig. 4). Predictor variables were selected using forward stepwise selection based upon reduction in AIC.

### Sperm whale population size

The population of sperm whales globally, *N*, was then calculated as follows:

1 $$N=\sum_{k}d\left(k\right)\cdot a\left(k\right),$$

where *a*{*k*} are the parameters of the regression; the summation is over *k*, the grid points; *d*(*k*) is the estimated sperm whale density at grid point *k* from the habitat suitability model; and *a*(*k*) is the area of the 1° cell centred on grid point *k*. Population estimates for other ocean areas (North Atlantic, North Pacific, Southern Hemisphere) were calculated similarly.

The CVs of these population estimates were calculated following the methodology in^8^, (although there is an error in Eq. (3) of^8^ such that the squareroot symbol covers both the numerator and denominator rather than just the numerator). The error due to uncertain density estimates for the different surveys is:

2 $$CV\left({D}_{T}\right)=\frac{\sqrt{\sum_{i}{\left(CV({n}_{i})\cdot {n}_{i}\right)}^{2}}}{\sum_{i}{n}_{i}}.$$

This is combined with the uncertainty in the extrapolation process (output from the linear models), CV(extrap.), to give an overall CV for the population estimate:

3 $$CV\left(N\right)=\sqrt{{CV({D}_{T})}^{2}+{CV(\mathrm{extrap}.)}^{2}.}$$

### Post-whaling trend in population size

We compiled a database of series of surveys producing population estimates of the same study area during the period 1978 (by which time most commercial sperm whaling had ceased) and 2022. Each series had to span at least 10 years, and all of the surveys in the series had to be comparable in terms of area covered throughout the time span. There also had to have been at least 3 surveys for a data set to be included.

The data consisted of the survey area, *A*, the estimated population in area *A* in year *y* (for multi-year surveys, *y* would be the midpoint of the data collection years), *n*_*E*_(*A*,*y*), and the provided CV of that estimate, CV(*n*_*E*_(*A*,*y*)). The data series used for these analyses are summarized in Table 3.

For each survey area, *A*, we calculated the trend in logarithmic population size, *r*(*A*), over time using weighted linear regression:

4 $${\text{Log}}\left( {n_{E} \left( {A,y} \right)} \right) \, \sim {\text{ constant}}\left( A \right) \, + r\left( A \right) \cdot y. \left[ {{\text{weight }} = { 1}/\left( {{1} + {\text{ CV}}\left( {n_{E} \left( {A,y} \right)} \right)} \right)^{{2}} } \right]$$

Table 3 also includes other published estimates of sperm whale population trends, from sighting rates or mark-recapture analyses of photoidentification data, with these estimates also having to span at least 10 years of data collection, and include data collected in three or more different years.

### Population trajectory

To examine possible trajectories of the global sperm whale population following the start of commercial whaling in 1712, we used a variant of the theta-logistic, a population model that has been employed in other recent analyses of the population trajectories of large cetaceans^45,63^. The theta-logistic model is:

5 $$n\left(y+1\right)=n\left(y\right)+r\cdot n\left(y\right)\left(1-{\left(\frac{n\left(y\right)}{n\left(1711\right)}\right)}^{\theta }\right)-f\left(y\right)\cdot c\left(y\right).$$

Here, *n*(*y*) is the population of sperm whales in year *y*, *r* is the maximum potential rate of increase of a sperm whale population, and θ describes how the rate of increase varies with population size relative to its basal level before whaling in 1711, *n*(1711). The recorded catch in year *y* is *c*(*y*) and *f*(*y*) is a correction for bias in recorded catches.

Whaling reduced the proportion of large breeding males^64^, likely disrupted the social cohesion of the females^3^, and may have had other lingering effects which reduced pregnancy or survival, and thus the rate of increase. Poaching has been found to reduce the reproductive output of African elephants, *Loxodonta Africana*, which have a similar social system to the sperm whales^3^, and this effect lingers well beyond the effective cessation of poaching^46^. There is some evidence for these effects of what we call “social disruption” on sperm whale population dynamics^20,46,65^. We added a term to the theta-logistic to account for such effects:

6 $$n\left(y+1\right)=n\left(y\right)\left[1+r\cdot \left(1-{\left(\frac{n\left(y\right)}{n\left(1711\right)}\right)}^{\theta }\right)-q\cdot \frac{\sum_{t=y-T}^{y}f(t)\cdot c(t)}{n\left(y-T\right)}\right]-f(y)\cdot c(y).$$

Here, $\frac{\sum_{t=y-T}^{y}f(t)\cdot c(t)}{n\left(y-T\right)}$ is the proportion of the population killed over the last *T* years, and *q* is the reduction in the rate of increase when almost all the whales have been killed. This reduction is modelled to fall linearly as the proportion killed declines to zero.

The global sperm whale population has some geographic structure^18^. Females appear to rarely move between ocean basins, and males seem to largely stay within one basin. Furthermore, sperm whaling was progressive, moving from ocean area to ocean area as numbers were depleted^4^. We model this by assuming *K* largely separate sperm whale subpopulations of equal size. Exploitation in 1712 starts in subpopulation 1 and moves to subpopulations 1 and 2 when the population 1 falls to α% of its initial value, and so on for the other ocean areas. The catch in each year in each area being exploited is pro-rated by the sizes of the different subpopulations being exploited. The population model for subpopulation *k*, which is one of the *K*_E_ subpopulations being exploited in year *y*, is:

7 $$n\left(k,y+1\right)=n\left(k,y\right)\left[1+r\cdot \left(1-{\left(\frac{n\left(k,y\right)}{n\left(k,1711\right)}\right)}^{\theta }\right)-q\cdot \frac{\sum_{t=y-T}^{y}C(k,t)}{n\left(k,y-T\right)}\right]-C\left(k,y\right),$$

where the estimated catch in year y in subpopulation k is given by: $C\left(k,y\right)=f(y)\cdot c(y)\cdot n(k,y)/\sum_{{k}^{\mathrm{^{\prime}}}=<{K}_{E}}n\left({k}^{\mathrm{^{\prime}}},y\right).$

Global catch data for sperm whales have been compiled by year for the periods 1762–1799^16^, 1800–1899^17^ and 1900–1999^6^ (Fig. 4). This catch record is not complete. There are no compiled data on sperm whaling between its inception in 1712^4^ and 1761. We filled this in by extrapolating a linear trend between the catch in 1711 (0 whales) and that in 1762 (351 whales). Catch records for the 18th and 19th Century are based on extrapolations from oil production records, and are not corrected for whales struck and lost that died, which will produce a downward bias in estimates of catches of the order of a few percent^16,17^. The 20th Century catch records are also likely downward biased by an unknown, but likely quite small, amount, primarily due to falsification of records^6^.

For any choice of population and exploitation parameters (*n*(1711), *r*, θ, {*f*}, *K*, α, *q*, *T*) a population trajectory can be calculated, giving a population size in 1993, *n*(1993). We have an estimate of the population in 1993 from the first part of this paper, but not in 1711. Thus, given values of the other parameters, the catch history {*c*}, and a value of *n*(1993), we found the value of *n*(1711) (using the “fzero” function of MATLAB2021b) so that the trajectory reached *n*(1993) in year 1993.

The best estimates, assumed distributions, and range of acceptable values for each of the parameters of the population model are given and justified in Table 4.

Using these parameter estimates, we ran the model in three ways: with the best estimate of each parameter; in a sensitivity analysis, using a range of values for each parameter in turn (the range given in Table 4) with the best estimates of the other parameters; and using random but reasonable values for all parameters (see Table 4 for assumed distribution of each) in each of 1,000 runs. Runs producing invalid (infinite or unreal) population estimates in 1711 were discarded.

The primary outputs of the population model were the estimated trajectory from 1711 to 2022, the current population (*n*(2022)), the estimated population in 1711 before the commencement of commercial whaling, and *n*(2022)/*n*(1940). Assuming three generations equals about 82 years^66^, *n*(2022)/*n*(1940) gives the proportional change in population size over 3 generations (the IUCN Red List measure for population trends). The proportional decline from pre-whaling numbers (i.e. 1711) to 2022 was also calculated.

## Supplementary Information

Supplementary Information.

## Data Availability

All data analysed during this study are included or referenced in this published article and its Supplementary Information.
