# Supplementary material for: Current global population size, post-whaling trend and historical trajectory of sperm whales
Source: Sci Rep. 2022 Nov 14;12:19468. doi: 10.1038/s41598-022-24107-7 (PMC9663694; doi:10.1038/s41598-022-24107-7)
Supplement: Supplementary file 1 — Supplementary Information. [file 41598_2022_24107_MOESM1_ESM.pdf]

SUPPLEMENTARY INFORMATION FOR:

Current global population size, post-whaling trend and historical trajectory of sperm whales

Hal Whitehead[1,*], and Megan Shin[1]

[1]Dalhousie University, Department of Biology, Halifax, B3H 4R2, Nova Scotia, Canada

*hwhitehe@dal.ca

SELECTION OF SURVEYS

For the population analysis, we selected surveys by: first using online literature searches (see Supplementary Table S1); second using the catalogue of surveys of US and adjacent waters in https://www.fisheries.noaa.gov/national/marine-mammal-protection/marine-mammal-stock-assessment-reports-species-stock; and third examining cited references in chosen papers. Usable surveys needed an estimate of sperm whale density or population size with CV for a well-defined study area. When data from the same survey were analyzed in two more papers (e.g. the Antarctic IDCR surveys), we used the most recent analysis. When two or more surveys covered more or less the same study area, but their data had not been combined into a single estimate, we selected as follows: a) the survey deemed most credible by the authors of the most recent paper describing the surveys (e.g. the case of the northern Atlantic where the 2007 survey was chosen over the 2015 survey as the correction for perception and availability biases for the latter was considered "anomalously low"[1]); b) if there was no such ranking, the survey with the most up-to-date methodology; c) if methodologies were similar, the most powerful survey by effort. Typically, this resulted in the choice of the most recent survey (exceptions included the IDCR Antarctic surveys where the 1985-1991 surveys had more survey effort than the 1991-1998 surveys[2]).

Two surveys (mid-Atlantic ridge and Bahamas) were omitted from further analysis because of their extremely low area coverage[3,4].

References

1. Pike, D. G. *et al.* Estimates of the abundance of cetaceans in the Central North Atlantic from the T-NASS Icelandic and Faroese ship surveys conducted in 2007. *NAMMCO Scientific Publications* **11**, (2019).

2. Branch, T. A. & Butterworth, D. S. Estimates of abundance south of 60°S for cetacean species sighted frequently on the 1978/79 to 1997/98 IWC/IDCR SOWER sighting surveys. *Journal of Cetacean Research and Management* **3**, 251–270 (2001).

3. Ward, J. A. *et al.* Passive acoustic density estimation of sperm whales in the Tongue of the Ocean, Bahamas. *Mar Mamm Sci* **28**, E444–E455 (2012).

4. Waring, G. T., Nøttestad, L., Olsen, E., Skov, H. & Vikingsson, G. Distribution and density estimates of cetaceans along the mid-Atlantic Ridge during summer 2004. *Journal of Cetacean Research and Management* **10**, 137–146 (2008).

5. NOAA. Digital relief of the Surface of the Earth. Data Announcement 88-MGG-02, National Digital Data Center. (1988).

6. Locarnini, R. A. *et al.* World Ocean Atlas 2005, volume 1: Temperature. *NOAA atlas NESDIS* **61**, (2006).

7. Antonov, J. I. *et al.* World Ocean Atlas 2005 volume 2: Salinity. *NOAA atlas NESDIS* **62**, (2006).

8. Garcia, H. E., Locarnini, R. A., Boyer, T. P. & Antonov, J. I. World Ocean Database 2005, Volume 3: Dissolved oxygen, apparent oxygen utilization, and oxygen saturation. *NOAA atlas NESDIS* **63**, (2006).

9. Garcia, H. E., Locarnini, R. A., Boyer, T. P., Antonov, J. I. & Levitus, S. World Ocean Atlas 2005, Volume 4: Nutrients (phosphate, nitrate, silicate). *NOAA atlas NESDIS* **64**, (2006).

Table S1. Search engines and keywords used for identifying suitable surveys.

Search Engine	Keywords	Timespan	Results
Web of science	TOPIC: (whale*, sperm whale*, "physeter macrocephalus", visual survey*) Refined by: TOPIC: (sperm whale*, visual survey*)	Timespan: All years. Indexes: SCI-EXPANDED, SSCI, A&HCI, CPCI-S, CPCI-SSH, ESCI.	24
Web of science	TOPIC: (cetacea*, visual survey*, population*, physeter macrocephalus, odontocete*,)	Timespan: All years.	1
Web of science	TOPIC: (visual survey*, population*, odontocete*,)	Timespan: All years.	7
Scopus	TITLE-ABS-KEY (sperm AND whale* OR whale* OR cetacea* OR physeter AND visual AND survey* OR population*)	Timespan: All years.	46
SciElo	cachalote*	Timespan: All years.	15
SciElo	sperm whale*	Timespan: All years.	16

Table S2. Measures that might predict sperm whale density.

Variable	Unit	Range of Collection	Source
Depth	m	-	[5]
Temperature	°C	Surface	[6]
Salinity	PSU	Surface	[7]
Dissolved O_2	ml/L	Surface	[8]
Phosphate	µg/L	Average, Surface, -500m, -1,000m	[9]
Nitrate	µg/L	Average, Surface, -500m, -1,000m	[9]
Chlorophyll A[1]	mg/m^3	Surface	https://neo.gsfc.nasa.gov/view.php?datasetId=MY1DMM_CHLORA
Eddy Kinetic Energy	m^2/s^2	-	https://www.aviso.altimetry.fr/en/data/products/sea-surface-height-products/global/gridded-sea-level-anomalies-mean-and-climatology.html

[1] Chlorophyll estimates used data from 2018-2020, and were log transformed after averaging.

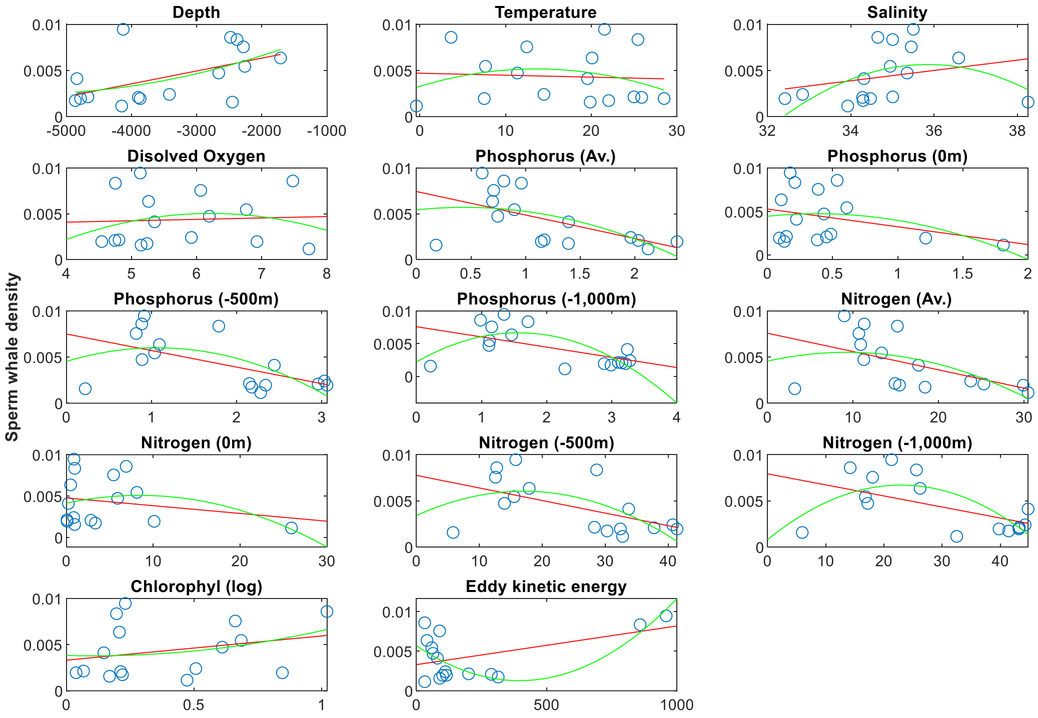

Figure S1. Relationship between mean values of the oceanographic predictor variables and sperm whale density across the different surveys, together with linear (red) and quadratic (green) best fit regressions. Sperm whale densities are in whales/km^2. Units of predictor variables are as in Table S2.

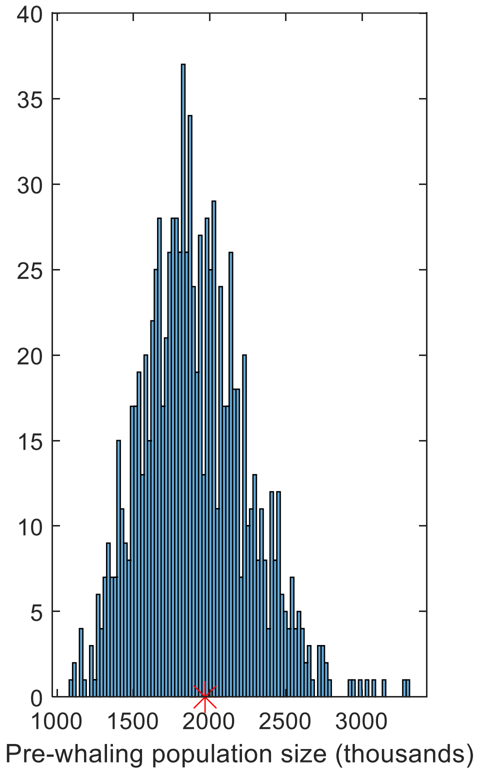

Figure S2. Distribution of estimates for pre-whaling (1711) global sperm whale population size (left), and 3-generation proportional decline (right), from 1,000 runs of the population model using randomly-chosen, but reasonable, parameters. *s show the outputs using the "best" parameter estimates.

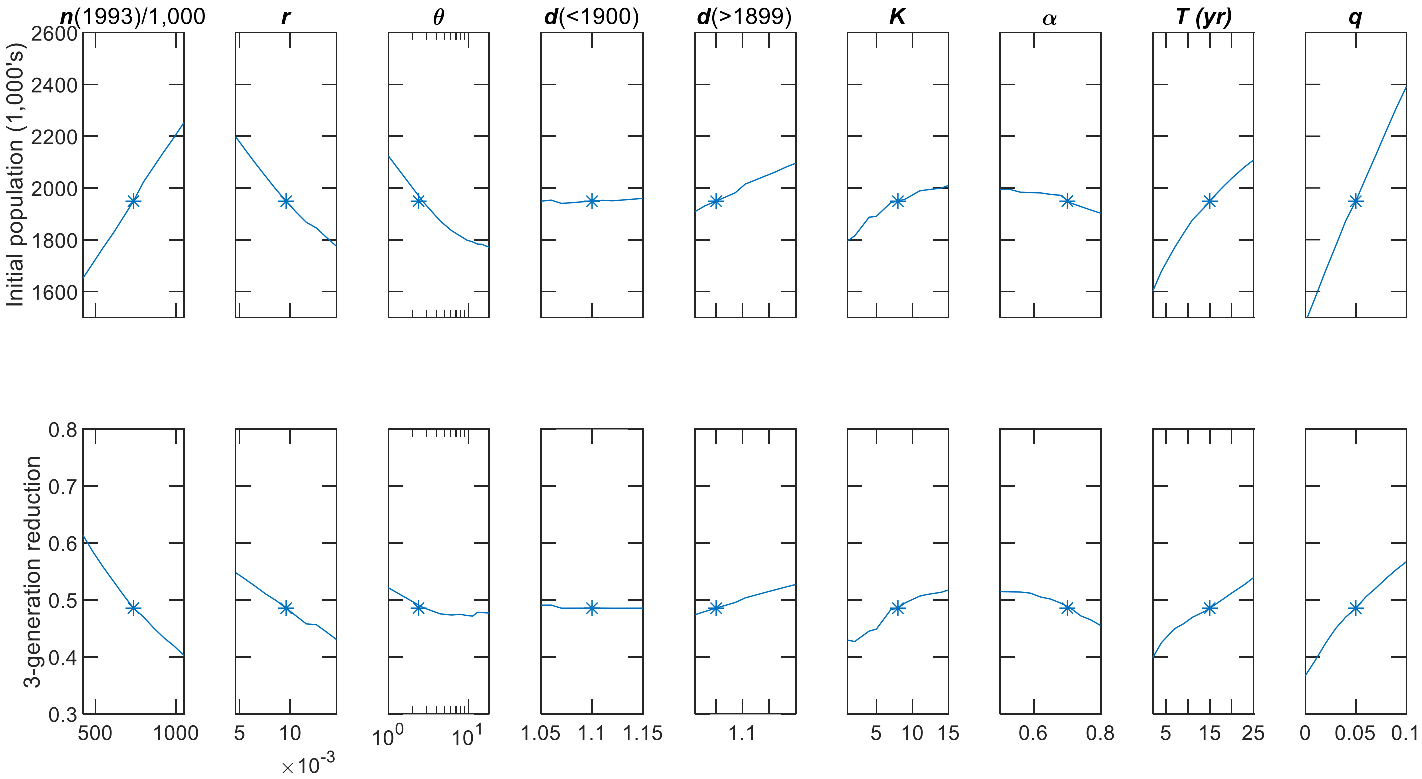

Figure S3. Sensitivity analysis. Change in outputs of model (initial population (in 1711) and 3-generation (1940-2022) decline) with chosen values of each of the input parameters, holding all other parameters at their "best" values. *'s are chosen "best" values for each parameter.
